# Insights into Advancements and Electrons Transfer Mechanisms of Electrogens in Benthic Microbial Fuel Cells

**DOI:** 10.3390/membranes10090205

**Published:** 2020-08-28

**Authors:** Mohammad Faisal Umar, Syed Zaghum Abbas, Mohamad Nasir Mohamad Ibrahim, Norli Ismail, Mohd Rafatullah

**Affiliations:** 1 Division of Environmental Technology, School of Industrial Technology, Universiti Sains Malaysia, Penang 11800, Malaysia; faisalumar@student.usm.my (M.F.U.); norlii@usm.my (N.I.); 2 Biofuels Institute, School of Environment, Jiangsu University, Zhenjiang 212013, China; 3 School of Chemical Sciences, Universiti Sains Malaysia, Penang 11800, Malaysia; mnm@usm.my

**Keywords:** bioremediation, renewable energy, organic pollutants, electrogens, wastewater

## Abstract

Benthic microbial fuel cells (BMFCs) are a kind of microbial fuel cell (MFC), distinguished by the absence of a membrane. BMFCs are an ecofriendly technology with a prominent role in renewable energy harvesting and the bioremediation of organic pollutants through electrogens. Electrogens act as catalysts to increase the rate of reaction in the anodic chamber, acting in electrons transfer to the cathode. This electron transfer towards the anode can either be direct or indirect using exoelectrogens by oxidizing organic matter. The performance of a BMFC also varies with the types of substrates used, which may be sugar molasses, sucrose, rice paddy, etc. This review presents insights into the use of BMFCs for the bioremediation of pollutants and for renewable energy production via different electron pathways.

## 1. Introduction

Different environmental pollutants, such as organic- and inorganic-based contaminants, remain a severe challenge to the sustainability of water resources [1,2]. This poses a serious threat to living organisms, including human beings and marine organisms [3]. Due to the depletion of natural water resources, there is an imbalance in the natural ecosystem, but simultaneously the commutability of renewable pure water resources has been enhanced. There is a plethora of potential sources of pollution in water bodies (e.g., oceans, lakes, rivers and reservoirs) stemming from human activity, and notably the chemical and oil filtration industries. The chemical substances emitted from these industries contain very harmful and potentially carcinogenic inorganic and organic pollutants [4]. These pollutants have a severe impact on living organisms and pose a serious threat to the environment.

Several techniques exist for the treatment of wastewater prior to irrigation, such as lagoon ponds, constructed wetlands, conventional wastewater treatment plants, membrane bioreactors and membrane filtration. Although these techniques have been shown to be effective, disadvantages remain, i.e., they require a large area for operation, along with high economic stability [5]. Recently, a novel approach was introduced for the treatment of wastewater: the microbial fuel cell. Microbial fuel cells (MFCs) are devices which utilize microbial activity to produce electricity from chemical energy stored in an organic substrate. Thus, MFCs are a promising technique for wastewater bioremediation and for generating electricity in an economical way.

Organic pollutant compounds are oxidized by microorganisms and the transfer of electrons to the anode of the MFC via exoelectrogens [6,7]. A new type of MFC, the benthic microbial fuel cell (BMFC), was designed to generate electricity from organic matter present in wastewater. As a result, like with MFCs, chemical energy is converted into electrical energy with exoelectrogens working as a catalyst, i.e., electrons (e^−^) and protons (H^+^) are released. In this way, a potential difference exists between the anode and cathode. Here, we present information regarding recent developments using exoelectrogens on the anode by direct and indirect processes.

## 2. Benthic Microbial Fuel Cell (BMFC)

There is a need for sustainable and clean energy sources to meet growing energy demands. In 2014, the global percentage of electricity generated via the consumption of fossil fuels was 66%; however, only 11% of this was utilized together with renewable energy [8,9]. Organic substrates are used as bio sediments, and they protect the microbial ecosystem in various regions and provide a suitable environment for the bioremediation of accumulated pollutants via the electron donor–acceptor mechanism [10]. Currently, physiochemical processes, such as dredging, ozonation and electrochemical degradation, are used for the bioremediation of pollutants. These techniques are effective but require a lot of energy and are costly, limiting their application. Usually, the accumulation of reductive substances and the lack of electron acceptors are the main limitations for the remediation of sediment under anaerobic conditions.

In recent years, microbial fuel cells (MFC) have been considered as an alternative, cheap approach to the bioremediation of toxic organic pollutants via power generation. Recently, BMFCs have attracted the attention of many researchers due to their nonaggressive and easily controllable nature. BMFCs consist of an anode, which is embedded in organic matter, and a cathode, which is placed in the overlying water. The air diffuser provides a constant supply of oxygen which plays a vital role in the transfer of electrons and protons from the anode to cathode via an external circuit, where electrons react with oxygen and produce water [11,12].

**Figure 1:**
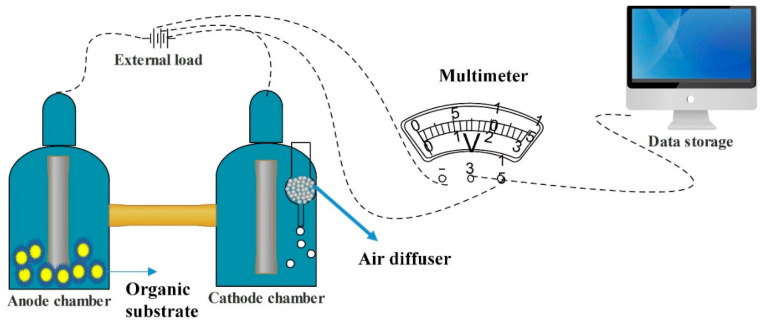
General prototype scheme of a benthic microbial fuel cell.

Reimers et al. [13] were the first to employ BMFCs; their approach included a platinum mesh for the anode and carbon fiber for the cathode. A unique feature of the BMFC is its membrane-less assembly; this is possible thanks to the boundary organic substrate used as a substrate, which itself acts as a pseudo membrane. Nowadays, many researchers are working on improving ecofriendly systems, including BMFCs [14]. The prototype of a double chamber BMFC is shown in Figure 1.

An air cathode in the overlaying water connected with a benthic-integrating anode is the most common BMFC model. In a saline environment, conductivity is normally high, so the overpotential limits the BMFC performance; this is not the case in freshwater [15]. Under the latter scenario, the efficiency of the anode decreases because of anodic contamination, i.e., the accumulation of waste substrate in the anodic region. BMFCs are usually restricted in terms of the proximity of the electrode by the naturally forming spatial separation of oxic and anoxic zones [16]. The tubular air cathode designs along with the cathodic fabric assembly structure suggest that only low-cost fabric would separate the electrodes. In this configuration, the cathode catalytic layer was exposed to air and would allow a hydrogen oxidation reaction [17]. However, as this setup requires long tubes for air exposure, the BMFC’s setup cannot operate in deep-water environment. If the BMFC can adapt the cathode carbon cloth, then embedded cathode in the organic substrate can also be used optionally [2]. In the simple design of the BMFC, though, electrodes can be constructed from both graphite felt or carbon cloth.

## 3. Degradation of Organic Matter by BMFC

**Figure 2:**
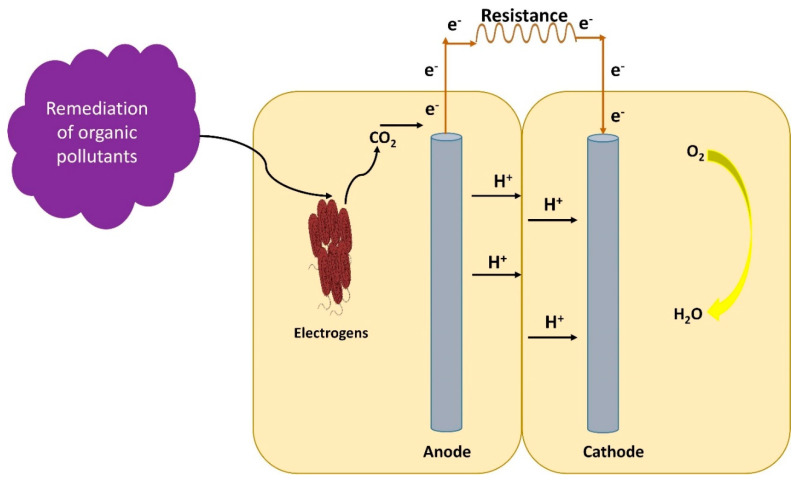
Overview of organic pollutants removal by benthic microbial fuel cell.

Like bio-electrochemical systems, BMFCs too have been shown to boost the organic compounds biodegradation, i.e., total petroleum hydrocarbons, total organic carbon, ignition loss and polycyclic aromatic hydrocarbons present in the wastewater, as shown in Figure 2. BMFC takes some time for the formation of a biofilm on the anode, which is the main requirement for the removal of the organic contents [18,19]. The anodic biofilm consists of two types of bacteria, the fermentative bacteria and the exoelectrogens. Fermentative bacteria are primarily involved in the complex organic matter hydrolysis and transform the products of hydrolysis into ethanol, H_2_, volatile organic acids and CO_2_ by acid-forming fermentation [20]. Ethanol, into which lactic acid can easily be converted, is volatile and readily escapes, allowing the reaction to proceed easily. CO_2_ is the other product, but is weakly acidic and even more volatile than ethanol. H_2_ is a substrate for methanogens and sulfate reducers, which keep the concentration of hydrogen low and favor the production of such an energy-rich compound, but hydrogen gas at a fairly high concentration can nevertheless be formed.

The metabolites of fermentative bacteria used by electrogenic bacteria as substrates, which produce electrons, CO_2_ and protons by oxidation, are shown in Equation (1). The protons are shifted to the overlying cathodic water and transfer few electrons towards the anode, which can be seen in Equation (2). These electrons are passed to the cathode through an external circuit and a redox reaction occurs that generates protons and dissolves oxygen, as mentioned in Equation (3) [21]. The existence of these electrodes has established a new microbial mechanism for metabolism, and to some degree it alters anodic microbial communities too. Recently, it has been reported that BMFCs alone cannot efficiently remove the organic pollutants. Wu et al. [18] reported that zero-valent iron (ZVI) has a high reducing ability (E0 = −0.44 V) and could react with the oxidizing contaminants. The hydroxyl radical formed through this method is a very durable oxidative degradation of bio-refractory organics, which allows for the common use of ZVI technology in the treatment of dyes, complexing agents, chlorinated organic compounds and preservatives. ZVI can also alter the metabolic pathways and redox capacity, regulate acidification and promote extracellular electron transfer. Estevezcanales et al. [22] cultivated *Geobacter sulfurreducense* with an iron-free substratum and found an abruptly reduced cytochrome *c*, which showed a limited capacity of outer membrane electrons transport. However, using ZVI alone, the desired effect cannot be guaranteed, particularly the final removal of certain refractory contaminants. The combined use of ZVI and BMFC technologies offers an enhanced substitute approach for eliminating organic contaminants.

Anode: (oxidation)

(1) $$a\left(\mathrm{OP}\right)+{\;\mathrm{bH}}_{2}O\to {\mathrm{cCO}}_{2}+{\;\mathrm{ne}}^{-}+{\mathrm{dH}}^{+}$$

Cathode: (reduction)

(2) $${\mathrm{eO}}_{2}+{\;\mathrm{dH}}^{+}+{\mathrm{ne}}^{-}\to {\mathrm{bH}}_{2}O$$

Overall reaction: (redox reaction)

(3) $$a\left(\mathrm{OP}\right)+{\;\mathrm{eO}}_{2}\to {\mathrm{cCO}}_{2}+{\;\mathrm{bH}}_{2}O$$

a = number of organic pollutants (OP) molecules, b = number of water molecules, c = number of carbon dioxide molecules, d = number of protons, e = number of oxygen molecules and n = number of electrons.

The removal of organic contaminants from BMFC is the foremost priority for organic contents remediation. Many hydrocarbons, such as those consisting of nitro and chlorine aromatic compounds, can be employed as substrates in BMFC. For bioremediation, these compounds need bioreduction [23]. The amalgamation of bioremediation and the electrochemical system forms a synergistic connection among electrodes and bacteria and enables the bioreduction of perchloroethane and polycyclic aromatic hydrocarbons. The in-situ generation of oxygen and hydrogen can be employed for intermediates reduction. The energy efficiency and removal of these organic compounds can be upgraded by direct electron transfer to electrodes from exoelectrogens or the inclusion of dechlorinating species [24]. There is a proportional relationship among power production and the degradation of these organic compounds. This closed-circuit BMFC creates the optimum environment for the degradation of organic compounds. This system could have a negative impact on BMFC microbes if not used properly. During the remediation of the organic compound in BMFC, some common issue are encountered, such as cathodic pH becoming alkaline and anodic pH becoming acidic via water electrolysis [25]. Unequal nutrients distribution in the chamber, like nitrate and phosphate, accumulating in the cathode chamber and ammonium accumulating in the anode chamber are other issues encountered during the remediation. These issues not only effect the performance of BMFC but also the biological clogging. These issue can be resolved by reversal of electrodes polarity and with proper water circulation. The degradation of organic compounds is also influenced by the competitive reactions with nitrate and sulphate [26].

## 4. Electron Transfer Mechanism by Electrogens

**Figure 3:**
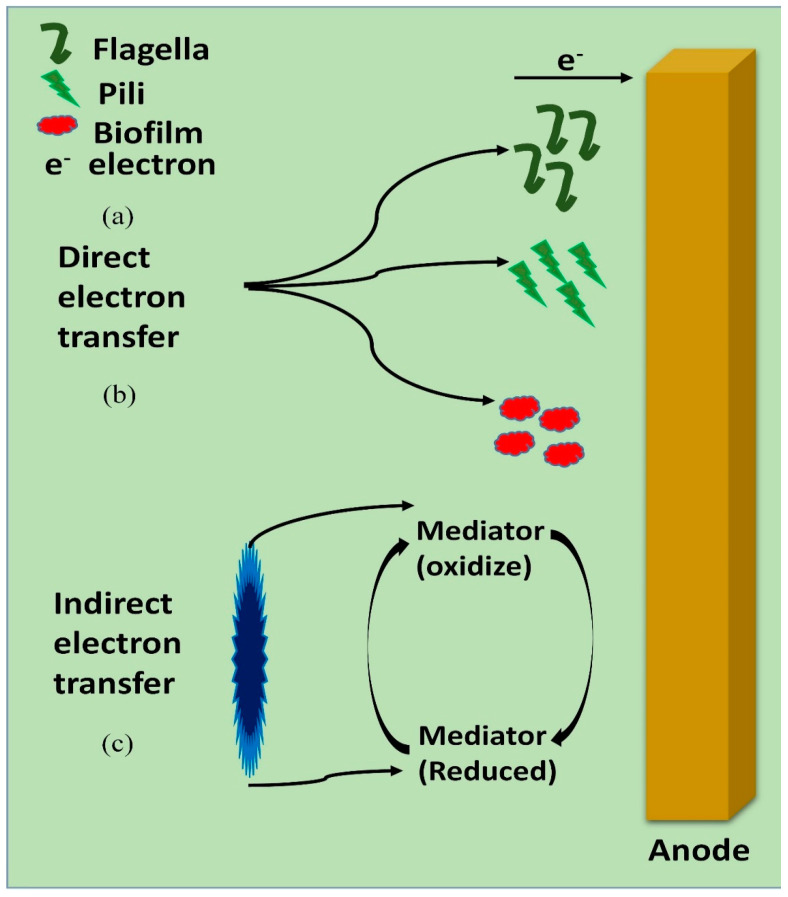
Proposed electron transfer mechanisms utilized direct electrons (**a**,**b**) and indirect electrons transfer (**c**) through electrogens using benthic microbial fuel cell.

The electrons transmission mechanism is essential in order to acquire a flawless knowledge for the application of BMFC at a large scale. In the anodic chamber of BMFC, organic substrates are reduced by microbes and transfer electrons to anodes, from where the electrons move to the cathode through external circuit to generate electricity [27]. Earlier, the microbes were exploited in the anodic chamber, but recently microbes are also exploited as biocathodes in the cathodic region to assist electrons transmission to the terminal electron acceptor (TEA) [28,29]. The power density, current density and coulombic efficiency can be measured by electron transfer rate. If the electrons transfer rate is higher than the electrons passing through the external circuit, more coulombic efficiency, power density and current density will be measured, leading to higher voltage production. The harvested bioenergy produced by the electron transfer towards electrodes from the respiration chain of electrogens is known as a new BMFC technology [30]. There are two means of electrons transfer in BMFC occupied by microbes: (i) direct electron transfer (direct contact between the microbes and the electrode surface) and (ii) indirect electron transfer (through the so-called electron mediators), as shown in Figure 3.

Recently, the application of electro-autotrophs in the Bioelectrochemical Systems (BES) has attracted the attention of researchers. The exoelectrogens use the electrodes or extracellular insoluble mineral as terminal electron acceptor (TEA), while electro-autotrophs accept the electrons from electrodes or solid compounds for CO_2_ reduction and produce multi-carbon compounds [31]. Gregory et al. first studied the electro-autotrophy in the *Geobacter*, which is a model exoelectrogen [32]. Most exoelectrogens are iron-oxidizing bacteria, which led to the hypothesis that dissimilatory iron-reducing bacteria can only accept the electrons from a cathode. Indeed, *Mariprofundus ferrooxydans* PV-1, *Acidithiobacillus ferrooxidans* and *Rhodopseudomonas palustris* have been selected as electro-autotrophs [33,34,35]. Furthermore, *Methanobacterium archaeon* strain IM1 and chemolithoautotrophic archea *Methanococcus maripaludis* were purified for electromethanogenesis with an electron donor (metallic iron) [36]. Many acetogenic bacteria like *Sporumosa acidovorans, Sporomusa silvacetica, Sporomusa sphaeroides, Sporumosa malonica, Moorella thermoacetica, Sporomusa ovate, Clostridium aceticum* and *Clostridium ljungdahlii* can also accept electrons from the cathode and reduce CO_2_ to organic acids [37]. Some sulphate-reducing autotrophs are believed to accept the electrons from cathode and generate hydrogen (H_2_) by reducing sulphate [38]. The cathodic electron consumption by bacteria causes anaerobic microbial-induced corrosion (MIC). The electro-autotrophs generate the corrosive hydrogen sulphide that results in chemically induced iron corrosion. The electro-autotrophs also stimulate the induced electrochemical corrosion by using cathodic hydrogen, which is generated by iron–water contact [39]. The benthic microbial fuel cells (BMFCs) were constructed for anaerobic exoelectrogenic enrichment, which separates the electrotrophic bacteria by opposing the anode to bio-cathode [40]. Recently, an MFC was developed initially with heterotrophic conditions that later alter with autotrophic conditions. After five batches of cultivation, the nonelectrochemical bacteria is dispersed into the liquid medium and only electro-autotrophs bacteria (*Geobacter*) were abundant in the MFC [41]. This electro-autotrophic process promotes the growth of exoelectrogens on the electrodes and reduces the number of nonelectrobiochemical bacteria, which finally increases the MFC’s efficiency. The electro-autotrophic enrichment of the bio-cathode offers a simplified approach to purify the bio-chemical from various inoculum sources. Initially, bacteria are grown heterotrophically on fructose, glycerol and glucose, followed by acclimation to the medium, and CO_2_ was provided as the sole electron acceptor [42]. The conventional cathode causes corrosion, denaturation and toxicity of material, but the bio-cathode is very cost-effective. The microbes must be chosen based on their capability to shift from heterotrophic to autotrophic metabolism. This pathway may help us to understand the metabolic pathways of different electron donors or acceptor microbes that have formed on bio-cathodes [43]. For the production of valuable organic and fuel commodities, pure culture was used because the diversified electro-autotrophs uptake the electrons from the negatively poised cathode for CO_2_ reduction with heavier coulombic efficiencies. The mixed cultures primarily generate the complex products and acetates, which maintains the microbial metabolism. The surfeit of products was generated by employing a viable BES system with pure culture of *Clostridium ljungdahlii*. Overall, though, very little research has been focused on the electro-autotrophs, particularly the electrons transfer pathways from cathode to bacteria and their applications.

### 4.1. Direct Electron Transfer

Electrons should interact between the outer membrane of the microbes and the electrode. The biofilm or electrically conductive nanowires (pili and flagella) were found over the surface of the anode formed by electrogens [44]. The transmission of electrons takes place by direct interaction without any external mediator through an external membrane’s cytochromes, nanowires and electron transport proteins in exchange with the microbial membranes. The external membrane’s cytochromes are bonded with nanowires and allow electrogens to use an electrode as an electron acceptor. Furthermore, the direct electron transfer mechanism fully depends on the electron transport proteins, and they play a crucial role in electron transfer from cytoplasm to mitochondrial membrane. The drawback of this mechanism is the very poor electron transfer rate, because the active sites of electron transmission are deeply embedded within the proteins [45]. Recently, many electrochemical bacteria like Shewanella and Geobacter nanowires have been folded for better electrons transmission [46,47]. For effective and fast electron transfer (coulombic efficiency), the nanowires form an electroactive layer instead of a normal single layer. *Geobacter* species are diverse in their current production ability; *Geobacter hydrogenophilus* and *Geobacter metallireducens* produced higher current densities (0.2 mAcm^−2^) than *Geobacter bremensis, Geobacter chapellei, Geobacter humireducens, Geobacter uraniireducen* and *Geobacter bemidjiensis*, which produced much lower current densities (0.05 mAcm^−2^) [48]. Some electrogesns reported direct electron transfer to electrodes, such as *Geobacter sulfurreducens* [49], *Rhodopseudomonas palustris* [50], *Anaeromyxobacter dehalogenansc* [51], *Geobacter lovleyi* [52], *Pseudomonas aeruginosa* [53], *Thermincola potens* [54], *Shewanella oneidensis* [55], *Geothrix fermentans* [56], *Thermincola carboxydophila* [57], *Shewanella putrefaciens* [58], and *Escherichia coli* [59].

Much less is known about direct electron transfer pathways in the electro-autotrophic bacteria. From the experiments, it is confirmed that the Fe species uptake the electrons secreted by the cathodic biofilm. It is also ventured that *c-type cytochromes*, which are crucial constituents of Fe extracellular electron uptake, also play a vital part in the electron transmission from cathode to electro-autotrophs [60]. In the light of this hypothesis, the metaproteomics and metagenomics of the diversified microbial community inhibit the self-regenerating biocathode’s effect whereby CO_2_ is reduced via *c-type cytochromes* directly acquiring electrons from the *Chromatiaceae* family and other proteins related with Fe(II) oxidation [61]. The Fe(0)-corroding sulphate reducing microbes (SRM) could also uptake the electrons [62], and this discovery paved the way for scientists to use these microbes in biocathodic BES employments. So, this negative metabolic character can be turned into a sustainable positive biotechnological solution. Up to now, though, only some pure SRM cultures are used as electro-autotrophs. The cathodic biofilm of *Desulfovibrio desulfuricans* ATCC 27774 exhibited electro-autotrophic characteristics at an employed cathodic potential (E_cath_) of −0.169 V vs. SHE. After 20 days, lactate was supplemented as the carbon source, not CO_2_, and a stable negative current was measured [63]. Consequently, other species (*Desulfovibrio caledoniensis* and *Desulfovibrio paquesii*) of the genus *Desulfovibrio* were used for H_2_ and cathodic current generation, employing lactate or bicarbonate as the carbon source and E_cath_ that enabled abiotic H_2_ evolution [64].

The pure cultures of *Desulfovibrio piger* and *Desulfosporosinus orientis* displayed the electro-autotrophic properties at E_cath_ = −0.31 V vs. SHE, which has a higher positive potential than the neutral redox potential of H_2_ evolution (E^0′^_H_+/H_2_ = −0.41 V vs. SHE) and gaseous CO_2_ supplemented as an inorganic source [65]. *Desulfovibrio piger* (SRM *Deltaproteobacterium*) is a H_2_-oxidizing, Gram-negative, nonspore-forming electro-autotroph. It could oxidize organic matter, like lactate, pyruvate ethanol and, partly, acetate. Before this, its autotrophic metabolism effect on CO_2_, was not reported for other *Desulfovibrio* species. *Desulfosporosinus orientis* (SRM class Clostridia) is an acetogenic, capable of executing anaerobic sulfate respiration, and is a spore-forming electro-autotroph. The broad range of energy sources, such as pyruvate, ethanol, formate, methanol, H_2_, Fatty acids, lactate CO and CO_2_, can be used by *D. orientis* [66]. It can use various TEAs, such as sulphite, sulphate, sulphur dioxide and thiosulfate [67].

In BES, for the first time *Desulfopila corrodens* strain IS4 was identified as an Fe(0)-corroding SRM [38]. By using an electron donor (metallic iron), this *Deltaproteobacterium* (Gram-negative) was quarantined from marine sediment. This strain performs very fast hydrogen generation and sulphate reduction by consuming iron as an energy source as compared to orthodox hydrogen-foraging *Desulfovibrio* species. In BES, by using CO_2_ as the growth substrate at E_cath_ = −0.4 V vs. SHE, direct electron uptake was accomplished [38]. Currently, *Desulfobacterium autotrophicum* HRM2 (sulphate reducing bacteria) is being reported as an electro-autotroph at E_cath_ = −0.5 V vs. SHE. This *Deltaproteobacterium*, secluded from marine mud, is a fully SRM oxidizer having both directional pathways (Wood-Ljungdahl) and relating to the *c*-Cyt rich group [68]. *D. autotrophicum* HRM2 showed a high coulombic efficiency (83 ± 6%) and a capacity for acetate bio-electro synthesis [69].

### 4.2. Indirect Electron Transfer

Indirect electron transfer does not require direct physical interaction between the microbes and electrons acceptors. The small molecules and soluble mediator are involved in the inducement of this electron’s transfer mechanism. In this mechanism, the electrons mediator enters into the microbes, where the electrons are extracted by a metabolic reaction of electrogens, and finally these electrons are transferred to an anode [70]. Initially, at the first BMFC operative phase, the presence of electron mediators was considered as important. The electron mediators auxiliary in the BMFC anodic chamber are produced by electrogens. Several types of species had been investigated, as the synthesis of self-mediators known as endo-electrogens mediators, such as phenazine and pyocyanin, could be secreted by *Shewanella* and *Pseudomonas* species [71]. The potential differences between several electron mediators and redox proteins were reported in many studies, which significantly affects the electron transfer efficiency of different species [72]. However, the tendency of electrons transfer is affected by different chemical compounds known as exoelectricigens mediators, such as anthracenedione, thionine, neutral red, humic acid, riboflavin and methylene blue [73,74,75]; both exo-electrogens and endo-electrogens are shown in Table 1. These electrogens are exploited to transfer the electrons from inside of the cell towards the electrode, and different microbes have a different capability to transfer electrons from cell to electrode.

Very little is known about the electron uptake by acetogens from the cathode. Currently, by using a genetic system, it is being confirmed that *Clostridium ljungdahlii* (Gram-positive) exhibits protons pumps that cause proton motive force, which is necessary for its growth with CO_2_ as a carbon source [111]. This gives clues about the energy conservation mechanism in the electro-autotrophic acetogens. In *Clostridium ljungdahlii*, the electron uptake mechanism is differently predicted, because it cannot synthesize quinones or *c*-type cytochromes [112]. By using genetic toolbox, the properties and electron uptake pathways of *Clostridium ljungdahlii* could be clearer, and also give information about the electron uptake pathways of many Gram-positive bacteria. The genomic sequence of acetogenic *Sporomusa ovata* (Gram-negative) is available now. Genes coding for type IV pili and *c*-type cytochromes are present in the genomic sequence, which are the two main parts of the extracellular electron transfer mechanism [113]. The *c*-type cytochromes are a precarious factor for the extracellular electron transfer mechanism in both electrotrophs and electrogenic types. In *Geobacter* spp., pili type IV are long strings that exhibit the metal-like conduction of long-range electron transfer. The gene coding for Ubiquinone also present in the genome of *Sporomusa ovate* is also crucial for the electron transfer pathway [114]. *Sporomusa ovate* has many extracellular electron transfer components, which proves that the electron uptake mechanisms of *Sporomusa ovata* are similar to those of other electrotrophic and electrigenic bacteria. *Sporomusa sphaeroides*-related acetogens showed direct electron transfer mechanisms. This showed that Gram-negative acetogens could use this strategy of electron transfer in different environments [115].

## 5. Performance of BMFC Affected by Organic Substrate

**Figure 4:**
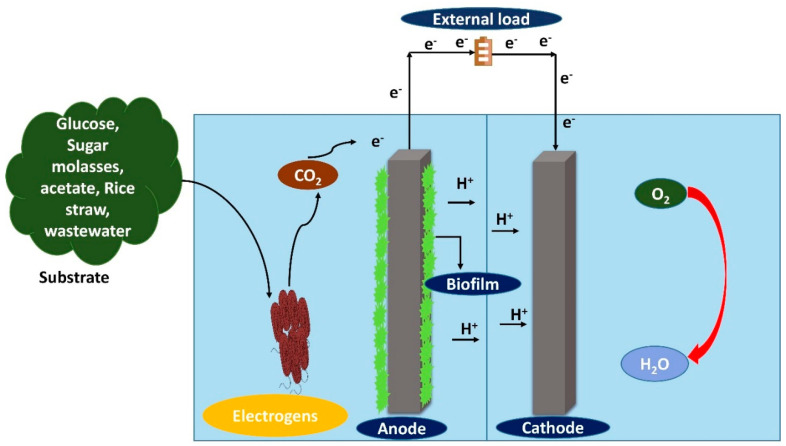
Representation of power generation by using organic contents as substrates by electrogens in a benthic microbial fuel cell.

In BMFC, the chemical reaction is replaced by a microbial reaction where the organic substrates are utilized as fuel for feeding the microbes and generating renewable energy. All these microorganisms that grow are nourished by varieties of substrates, which include simple carbohydrates or polysaccharides, amino acids, organic acids, cellulose and lignocellulose [95]. Marine sediments and aqueous ones were also employed in BMFC as a substrate [116]. The substrate not only facilitates the microbes in producing the biofilm on the surface of the anode, but is also designed to increase the performance of the BMFC by producing higher coulombic efficiency and power density [117]. Moreover, the diverse substrate processes fully depend on the biodegradability factor. The power density of BMFC is directly proportional to the quantity of organic contents in the organic substrate and the biodegradation by electrogens of the microorganism [118]. The mechanism of organic substrate degradation through electrogens using BMFC is shown in Figure 4.

Hassan et al. [119] studied the different organic substrates (glucose, fructose and sucrose) used in BMFC. Wang et.al. [120] developed a BMFC to generate a power density of about 12.7 mW/m^2^ using an electron-mediating agent at pH 4, with the help of acidophilic bacterium, *Acidiphilium cryptum*, utilizing glucose as the organic substrate. The pure bacterial strain *Brevibacillus borstelensis* STRI1 produced a power density of about 188.5 mW/m^2^ by using sugarcane molasses as the organic substrate [119]. The rice straw was also used as an organic substrate to generate a power density of about 293.33 ± 7.89 mW/m^2^ [121]. The existing literature reveals the different kinds of organic waste being used as organic substrates, with their corresponding capacities for power density generation by electrogens, as shown in Table 2.

In the BMFC, various kinds of substrates could be employed; these substrates can be starch, petroleum-based compounds, cysteine, glucose, dairy-based, acetate, molasses, glutamic acid, food-based wastewater, river water and vegetable-based. The substrate selection is based on their biodegradability behaviors. The power production by BMFC depends upon the degradation rate by the bacteria and the quantity of organic contents in the substrates [147]. In BMFCs, there is a continuous generation of power which is impeded by access to nutrients in the anodic media. The nutrients in BMFCs are regularly supplied with fresh matter from the decay of microbes and animals, giving the BMFC an indefinite life span in theory [148]. In the BMFC, one biodegradable fuel was also the bio-battery, but with this the power generation ultimately drops with time. Some substrates only support a single form of organic material. Different types of chitin were also used in BMFC anode as substrates. Chitin 80 and chitin 20 produced optimum power of about 84 ± 10 and 76 ± 25 mW/m^2^, respectively. The internal resistances of chitin 80 and chitin 20 were 650 ± 130 and 1300 ± 440, respectively. The electricity production could be enhanced by using substrates of precise size, and slowly degradable substrates. The substrates of precise size enhance the degradation surface area, and the slowly degradable substrates enhance the power production duration [146].

## 6. Conclusions

BMFC is a novel bio-technique that may be a potential solution to the two main problems, namely pollutants bioremediation and sustainable energy production. These BMFCs will open new possibilities for sustainable, cost-effective and controllable ways to generate power and bioremediate toxic pollutants. For power generation, there are two main routes of electron transfer: direct electron (physical contact between electrogens and anode) and indirect electron (conductive pili and flagella) transfer from the electrogens towards the anode of BMFC. The performance of BMFC depends on the use of different organic matters as the substrate. The novel BMFC technology will be encouraging for in situ pollutants bioremediation. The challenges of BMFCs will be addressed jointly by the efforts of scientists from many fields, such as environmental sciences, biotechnology, electrochemistry, electrical engineering, biology and material sciences.

## Figures and Tables

**Table 1:** Performance of BMFC configuration through exoelectrogens and endoelectrogens with respect to power density.

Microorganisms	External Mediator	Power Density (mW m^−2^)	Configurations	Type of Electrons Transfer Mechanisms	References
**Exoelectrogens microorganisms**					
*Shewanella oneidensis* strain 14063	1–amino–2–Napthol	>40	Single chamber	Direct transfer	[76]
*Shewanella oneidensis*	Anthraquinone–2,6–disulfonate (AQDS)	24	Double chamber	Direct transfer	[77]
*Klebsiella pneumoniae*	HNQ as mediator biomineralized manganese as electron acceptor	_	_	Direct transfer	[78]
*Pseudomonas species*	phenazine–1–carboxamide	_	_	Indirect transfer	[79]
*Pseudomonas aeruginosa*	phenazine compounds	3322 ± 38	Single chamber	Direct transfer	[80]
*Cellulomonas fimi*	anthraquinone–2,6–disulfonate	38.7	Double chamber	Direct transfer	[81]
*Lactococcus lactis*	Riboflavin, flavins	_	Double chamber	Direct transfer	[82]
*Geobacter sulfurreducens*	*c*–*Cytochrome z, type IV pili*	3147	Double chamber	Direct transfer	[83]
*Shewanella oneidensis* DsP10	Anthraquinone–2,6–disulfonate (AQDS)	5000	Double chamber	Direct transfer	[77]
*Rhodopseudomonas palustris DX-1*	*c*–*Type cytochromes*	2720	Single chamber	Indirect transfer	[49]
*Desulfovibrio desulfuricans ATTC*	*c*–*Type cytochromes*	1580	Single chamber	Indirect transfer	[84]
*Geobacter metallireducens*	*c*–*Type cytochromes, OmcE and OmcB*	450	Single chamber	Indirect transfer	[85]
*Desulfuromonas acetoxidans*	*c*–*Type cytochromes*	2000	_	Indirect transfer	[13]
*Klebsiella pneumonia*	*2,6–Di*–*tert*–*butyl*–*p*–*benzoquinone*	199	_	_	[86]
*Desulfovibrio alaskensis*	*Transmembrane complexes, tetraheme cytochrome C3*	_	_	_	[87]
*Pseudomonas aeruginosa*	Phenazine–1–carboxamide, pyocyanin	4300	_	_	[88]
*Thermincola ferriacetica*	Anthraquinone–2,6–disulfonate	12,000	Single chamber	_	[89]
*Shewanella putrefaciens*	c–Type cytochromes including OmcA, MtrC, FAD transporter	492	Double chamber	Indirect transfer	[90]
*Dechlorospirillum anomalous strain WD*	*Anthraquinone*–*2,6*–*disulfonate hydrogen*	30	_	Direct transfer	[91]
*Geobacter lovleyi*	*Methyl viologen*	480	_	Indirect transfer	[92]
*Chlorella vulgaris*	*Methyl viologen, methylene blue*	30	Single chamber	Indirect transfer	[91]
*Pseudomonas* sp.	*Methylene blue*	979	Single chamber	Indirect transfer	[93]
**Endo** **electrogens microorganism**					
*Rhodoferax ferrireducens*	_	158	Double chamber	Direct transfer	[94]
*Klebsiela pneumoniae* strain L17	_	34.77	Double chamber	Direct transfer	[95]
*Nocardiopsis* sp. KNU (strain), *Streptomyces enissocaesilis* KNU (K strains)	_	162 145	Double chamber	Direct transfer	[96]
*Rhodoferax ferrireducens*	_	_	Double chamber	Direct transfer	[97]
*Escherichia coli* strain K-12	_	215	Single chamber	_	[98]
*Shewanella oneidensis*	_	_	Single chamber	_	[99]
*Pseudomonas aeruginosa*	_	136 ± 87	Single chamber	_	[100]
*Cellulomonas fimi*	_	0.74 ± 0.07	Single chamber	Indirect transfer	[101]
*Leptothrix discophora SP-6*	_	70	_	Indirect transfer	[102]
*Acinetobacter calcoaceticus*	_	110	_	Indirect transfer	[50]
*Escherichia coli*	_	3390	_		[103]
*Winogradskyella poriferorum*	_	40	_	Indirect transfer	[104]
*Pseudomonas fluorescens*	_	210	Double chamber	Direct transfer	[105]
*Citrobacter* sp.	_	205	Double chamber	Indirect transfer	[106]
*Lysinibacillus sphaericus*	_	850	Double chamber	Direct transfer	[107]
*Dechloromonas* sp.	_	300	Double chamber	Indirect transfer	[108]
*Arthrospira maxima*	_	100	Double chamber	Direct transfer	[109]
*Coriolus versicolor*	_	3200	Single chamber	Indirect transfer	[110]

**Table 2:** Different substrates used in the BMFCs with corresponding power densities.

Waste Substrate	Electircigens	Power Density (mW/m^2^)	Configurations	Type of Electrons Transfer Mechanisms	References
Glucose	*Acidiphilium cryptum*	12.7	Single chamber	Direct transfer	[120]
Cellulose	*Enterobacter cloacae*	5.4 ± 0.3	Double chamber	Direct transfer	[122]
Lactate	*Shewanella oneidensis* MR-1	0.3 × 10^−2^	Single chamber	Indirect transfer	[123]
Lactate	*Geobacter sulfurreducens*	52 ± 4.7	-	Indirect transfer	[124]
Glucose	*Escherichia coli*	228	-	Indirect transfer	[125]
Malt extract	*Enterobacter cloacae*	9.3	-	Indirect transfer	[126]
*Cellulose*	*G. sulfurreducens* and *C. cellulolyticum*	83	Single chamber	Indirect transfer	[127]
*Wheat straw*	*Acidithiobacillus caldus*	123	Single chamber	-	[128]
Molasses	*B. borstelensis* STRI1	185.5	Single chamber	-	[119]
Sophorolipid with glucose and PBS	*Pseudomonas aeruginosa*	15.29	Single chamber	-	[129]
Glucose, fructose, and sucrose	*Saccharomyces cerevisiae*	72.77	Single chamber	-	[130]
Glucose in synthetic wastewater	_	1313	Double chamber	Direct transfer	[131]
xylose	*Geobacter sulfurreducens Escherichia coli,*	590	Double chamber	Direct transfer	[132]
Synthetic wastewater	*α*–*Proteobacteria*, *β*–*Proteobacteria*, *γ*–*Proteobacteria*	70	Double chamber	-	[133]
Sodium Fumarate	*Geobacter sulfurreducens*	_	Single chamber	-	[134]
Glucuronic acid	*Rhodococcus sp. and Paracoccus sp.*	2770	Double chamber	-	[135]
Xylose	*Clostridium spp. and Comamonas spp.*	1241	_	Direct transfer	[136]
Acetate	_	1430	_		[137]
Ethanol	Proteobacterium sp., Azoarcus sp. and Desulfuromonas sp.	40	_	Indirect transfer	[138]
Synthetic wastewater with molasses and urea	_	2.9	Single chamber		[139]
Cysteine	*Shewanella affinis*	39	_	_	[140]
Starch	*Clostridium butyricum or Clostridium beijerinckii*	_	_	_	[141]
Dye-containing wastewater in microbial desalination	*Bacillus subtilis, Aeromonas hydrophila subsp. hydrophila*	2.86	_	_	[142]
Rice straw	*Cellulose-degrading bacteria*	146	_	_	[121]
Coconut husk retting	*Ochrobactrum sp.*	362	Double chamber	Indirect transfer	[143]
Agriculture wastewater	*Shewanella oneidensis*	13	Double chamber	Indirect transfer	[144]
Rice paddy	*Geobacteraceae*	_	Double chamber	Indirect transfer	[145]
Chitin	*Bacillus circulans*	1.742	Double chamber	Indirect transfer	[146]
